# Determinants of Futile Recanalization and the Influence of Age After Endovascular Therapy for Acute Ischemic Stroke: A Retrospective Single-Center Cohort Study

**DOI:** 10.3390/medicina62102036

**Published:** 2026-10-21

**Authors:** Zeynep Tanriverdi, Eren Mingsar, Ibrahim Emik, Hatice Sabiha Ture, Omur Balli, Yesim Beckmann

**Affiliations:** 1 Department of Neurology, Izmir Ataturk Training and Research Hospital, Karabaglar, 35360 Izmir, Turkey; ibrahimemik@gmail.com (I.E.); sabihature@yahoo.com (H.S.T.); ybeckmann@gmail.com (Y.B.); 2 Department of Critical Care Medicine, Izmir Ataturk Training and Research Hospital, Karabaglar, 35360 Izmir, Turkey; mingsar@hotmail.com; 3 Department of Interventional Radiology, Izmir Ataturk Training and Research Hospital, Karabaglar, 35360 Izmir, Turkey; omurballi@gmail.com

**Keywords:** acute ischemic stroke, endovascular therapy, large-vessel occlusion, futile recanalization, functional outcome

## Abstract

*Background and Objectives*: Functional independence is not always achieved after successful recanalization with endovascular therapy (EVT) for acute ischemic stroke, a phenomenon termed futile recanalization (FR). We investigated factors independently associated with FR and explored whether these associations differed by age. *Materials and Methods*: In this retrospective single-center study, 231 consecutive patients treated with EVT between January 2021 and December 2024 were analyzed. FR was defined as a modified Rankin Scale score > 2 at 90 days despite a modified Thrombolysis in Cerebral Infarction (mTICI) grade of 2b–3. Model 1 included all patients with successful recanalization. Model 2 was restricted to patients with anterior circulation infarction and assessable imaging and additionally included the Alberta Stroke Program Early CT Score (ASPECTS) and collateral status. Age interactions were tested. *Results*: Recanalization was successful in 191 patients (82.7%), of whom 114 (59.7%) had FR. In Model 1 (*n* = 191), admission National Institutes of Health Stroke Scale (NIHSS) score (odds ratio [OR], 1.29 per point; 95% confidence interval [CI], 1.18–1.42), admission glucose (OR, 1.01; 1.00–1.02), and puncture-to-recanalization time (OR, 1.02; 1.00–1.03) were independently associated with FR. In Model 2 (*n* = 146), sufficient collateral status (OR, 0.19; 0.08–0.47), ASPECTS (OR, 0.60; 0.39–0.91), NIHSS score, neutrophil-to-lymphocyte ratio (NLR), dyslipidemia, and procedural duration were independently associated with FR, whereas glucose was not retained; Model 2 had an area under the curve of 0.88. Age ≥ 80 years was not independently associated with FR; the only significant interaction was with glucose in Model 1. *Conclusions*: Stroke severity and procedural duration were independently associated with FR in both models. Imaging variables contributed to prognostic assessment in anterior circulation infarction. Age ≥ 80 years was not independently associated with FR. The NLR association and age-stratified findings remain exploratory.

## 1. Introduction

Stroke remains one of the leading causes of mortality and long-term disability worldwide [1]. Endovascular therapy (EVT) has become the standard of care for patients with acute ischemic stroke (AIS) caused by large vessel occlusion (LVO). The most recent clinical guidelines emphasize that EVT significantly improves functional outcomes while reducing mortality in eligible patients with AIS [2].

Although successful recanalization rates following EVT approach 90%, only approximately half of patients achieve functional independence after treatment [3]. Moreover, despite successful recanalization, a substantial proportion of patients (60–70%) continue to experience poor functional outcomes at 3 months, and nearly one-third die during their hospital stay [4]. These findings indicate that favorable clinical outcomes depend on factors beyond successful vessel recanalization alone. Several variables have been shown to influence prognosis, including advanced age, comorbid conditions, the interval between stroke onset and recanalization, and the occurrence of hemorrhagic transformation [5,6,7,8,9,10].

Identifying the determinants of futile recanalization (FR) in patients undergoing EVT is essential for improving prognostic stratification and for developing therapeutic strategies that may optimize patient management and clinical outcomes. Advanced age is accompanied by reduced collateral reserve, a higher burden of comorbidity and frailty, and a different distribution of stroke etiologies; the prognostic weight of individual predictors may therefore not be uniform across the age spectrum, a question that has rarely been addressed in previous FR cohorts. Therefore, the present study aimed to identify the factors independently associated with FR in patients with AIS who underwent EVT and, as a secondary exploratory objective, to examine whether the variables associated with poor 90-day functional outcome differ between patients aged <80 and ≥80 years.

## 2. Materials and Methods

### 2.1. Study Design and Population

This was a retrospective, single-center cohort study, reported in accordance with the Strengthening the Reporting of Observational Studies in Epidemiology (STROBE) statement. Consecutive patients who underwent EVT for AIS due to LVO between January 2021 and December 2024 were retrospectively evaluated. A total of 253 adult patients (≥18 years) who presented to the emergency department with AIS and underwent EVT were initially screened. Twenty-two patients were excluded because clinical data or 90-day follow-up were unavailable. A total of 231 patients were included in the study. The patient selection process and the samples used in each analysis are shown in Figure 1.

### 2.2. Clinical and Radiological Data Collection

Demographic characteristics and vascular risk factors, including hypertension (HT), diabetes mellitus (DM), atrial fibrillation (AF), dyslipidemia, and chronic kidney disease (CKD), were recorded. Information regarding pre-stroke antiplatelet and anticoagulant therapy, as well as the length of hospital stay, was obtained from the medical records.

Frailty was assessed using the 5-factor modified Frailty Index (mFI-5), which incorporates functional dependence (modified Rankin Scale [mRS]), HT, DM, chronic obstructive pulmonary disease, and congestive heart failure [11,12]. Pre-stroke functional status was assessed retrospectively from hospital discharge summaries and outpatient records and documented using the mRS.

Stroke etiology was classified according to the Trial of Org 10172 in Acute Stroke Treatment (TOAST) criteria [13]. Occlusion sites were categorized as the internal carotid artery, middle cerebral artery (M1 or M2 segments), or basilar artery. Patients were further classified according to infarct location as anterior or posterior circulation stroke. Treatment strategy was categorized as direct EVT or bridging therapy consisting of intravenous recombinant tissue plasminogen activator (IV rtPA) followed by EVT.

The Alberta Stroke Program Early CT Score (ASPECTS) was calculated for anterior circulation infarcts before EVT [14]. Collateral circulation was assessed only in patients with anterior circulation stroke using preprocedural computed tomography angiography of sufficient image quality. Collateral status was graded according to the modified Tan score, with collateral filling > 50% classified as sufficient and ≤50% as insufficient collateral circulation [15]. An interventional radiologist blinded to the clinical data retrospectively reviewed baseline non-contrast CT and CT angiography (CTA) images. ASPECTS and collateral grading were used only for anterior circulation infarction. Baseline images were unavailable for 34 patients in this group because studies performed at referring institutions or early in the study period could not be retrieved from the archive, a reason unrelated to patient outcome. Both measures were therefore available for 172 of 206 patients with anterior circulation infarction, including 146 of the 171 who achieved successful recanalization. Stroke severity at admission was assessed using the National Institutes of Health Stroke Scale (NIHSS) [16].

### 2.3. Procedural Variables and Outcome Measures

Procedural time intervals, including onset-to-IV rtPA time, onset-to-groin puncture time, and puncture-to-recanalization time, were recorded.

Post-procedural intracranial hemorrhage (ICH) was classified according to the European Cooperative Acute Stroke Study criteria [17]. Symptomatic intracranial hemorrhage (sICH) was defined as any intracranial hemorrhage on follow-up imaging associated with neurological deterioration of ≥4 points on the NIHSS. Patients who underwent decompressive hemicraniectomy were also identified, and this variable is reported as decompressive surgery in the tables. In-hospital mortality was defined as death from any cause during the index hospitalization.

The degree of reperfusion following EVT was assessed using the modified Thrombolysis in Cerebral Infarction (mTICI) scale. Successful recanalization was defined as an mTICI grade of 2b–3 [18]. An interventional radiologist independently reviewed the final angiographic runs and assigned the mTICI grade while blinded to the procedural report and functional outcome. The database recorded grades 0, 1, 2a, 2b, and 3; grade 2c was not recorded separately. Patients were grouped as mTICI 0–2a, 2b, or 3.

FR was defined as successful angiographic recanalization (mTICI 2b–3) despite an unfavorable functional outcome at 90 days (mRS > 2).

### 2.4. Laboratory Assessment

Admission laboratory parameters, including the neutrophil-to-lymphocyte ratio (NLR), blood glucose, and serum creatinine levels, were recorded.

### 2.5. Functional Outcome

Functional outcome at 90 days was assessed using the mRS [19]. A favorable functional outcome was defined as an mRS score of 0–2, whereas an unfavorable functional outcome was defined as an mRS score > 2. An intensive care specialist assessed the 90-day mRS from discharge summaries and outpatient records while blinded to procedural and angiographic data. A 90-day mRS score was available for all 231 patients included in the analysis; no patient was lost to follow-up.

### 2.6. Ethics Approval and Informed Consent

The study was conducted in accordance with the principles of the Declaration of Helsinki and was approved by the Ethics Committee of İzmir Atatürk Training and Research Hospital (Ethics Approval Number: 0817, Date: 15 January 2026). The requirement for patient consent was waived because the study retrospectively analyzed anonymized data collected during routine care.

### 2.7. Statistical Analysis

All analyses were performed with IBM SPSS Statistics, version 26.0 (IBM Corp., Armonk, NY, USA). The distribution of continuous variables was assessed with the Shapiro–Wilk test and the homogeneity of variances with the Levene test. Continuous variables are presented as mean ± standard deviation or as median [interquartile range, IQR] according to their distribution, and categorical variables as counts and column percentages. Two-group comparisons of continuous variables were performed with the independent-samples *t*-test when the data were normally distributed and with the Mann–Whitney U test when they were not; categorical variables were compared using Pearson’s chi-square test or Fisher’s exact test when expected cell counts were <5. Comparisons among more than two independent groups were performed with one-way analysis of variance (ANOVA) for normally distributed data and with the Kruskal–Wallis test otherwise; significant Kruskal–Wallis results were followed by Dunn’s pairwise comparisons with Bonferroni adjustment. Effect sizes were reported alongside *p* values: Cramér’s *V* (equivalent to φ for 2 × 2 tables) for categorical variables, *r* = |*Z*|/√*N* for the Mann–Whitney U test, and η2H = (*H* − *k* + 1)/(*n* − *k*) for the Kruskal–Wallis test. Values of approximately 0.10, 0.30 and 0.50 for *V* and *r*, and 0.01, 0.06 and 0.14 for η2H, were interpreted as small, medium, and large effects, respectively [20].

Binary logistic regression was used to identify the factors independently associated with FR among patients with successful recanalization. Model 1 included clinical, laboratory, and procedural variables for all 191 patients with successful recanalization. Model 2 additionally included the imaging variables (ASPECTS and collateral status) and was restricted to the 146 patients with anterior circulation infarction and assessable baseline CT and CTA; 20 patients with posterior circulation infarction, in whom these measures are not applicable, and 25 patients with anterior circulation infarction whose baseline images could not be retrieved were excluded from Model 2 (Figure 1). The primary analyses were complete-case analyses. Variables with *p* < 0.20 in univariable analysis of each model sample were entered together into the multivariable analysis, followed by backward Wald selection (removal criterion, *p* > 0.10). LKW-to-needle time was excluded from multivariable modeling because it applied only to patients receiving IV thrombolysis (*n* = 90). Multicollinearity was assessed with the variance inflation factor; all values were <5, with no evidence of substantial multicollinearity. Model fit was assessed with the Hosmer–Lemeshow goodness-of-fit test and the Nagelkerke *R*^2^. Odds ratios (OR) with 95% confidence intervals (CI) were used to report the size of the associations.

Sensitivity analyses examined the robustness of the multivariable results. To examine the influence of vascular territory, Model 1 was refitted after restricting the sample to patients with anterior circulation infarction (*n* = 171). To separate changes related to the imaging variables from those related to sample restriction, Model 1 was refitted in the Model 2 sample (*n* = 146). To investigate missing imaging data, anterior circulation patients with and without assessable imaging were compared, and Model 2 was refitted in all 171 patients with anterior circulation infarction after multiple imputation of ASPECTS and collateral status by chained equations (predictive mean matching; 40 imputed data sets, 10 iterations each; the imputation model included the outcome and all candidate predictors), with estimates pooled according to Rubin’s rules. Effect modification by age was explored by adding age group (≥80 vs. <80 years) and its product term with each retained predictor to the final models, and the interaction terms were tested with likelihood-ratio tests.

Receiver operating characteristic (ROC) curves assessed discrimination by individual variables independently associated with FR and by the predicted probabilities from the final logistic regression models. Areas under the curve (AUC) with 95% CIs were calculated with DeLong’s method, AUCs of correlated ROC curves were compared with DeLong’s test, and optimal cutoff values were determined with the Youden index. Model 2 was internally validated with 500 bootstrap resamples in which the entire variable selection procedure was repeated, yielding an optimism-corrected AUC and a calibration slope. A nomogram derived from Model 2 estimated the probability of FR for individual patients. Sensitivity analyses, multiple imputation, DeLong tests, bootstrap validation, and all figures (flow diagram, forest plot, ROC curves, nomogram, calibration plot, and stacked percentage bar charts) were produced with Python, version 3.11 (Python Software Foundation, Beaverton, OR, USA), using statsmodels 0.15, scikit-learn 1.8, and matplotlib 3.10. Apart from the Bonferroni-adjusted post hoc comparisons, no adjustment for multiple comparisons was applied; therefore, secondary, subgroup, and age-stratified analyses should be regarded as exploratory. A two-sided *p* value < 0.05 was considered statistically significant.

## 3. Results

The study cohort consisted of 231 patients with a mean age of 67.7 ± 13.7 years (range, 21–93 years), of whom 118 (51.1%) were male. Anterior circulation stroke was the predominant infarct subtype, accounting for 89.2% (*n* = 206) of all cases. The most frequent occlusion site was the M1 segment of the middle cerebral artery (*n* = 118). HT was the most common vascular risk factor (61.5%, *n* = 142). According to the TOAST classification, large artery atherosclerosis was the most frequent stroke etiology overall, whereas cardioembolic stroke was significantly more common in patients aged ≥ 80 years (*p* = 0.045). The baseline demographic, clinical, radiological, and laboratory characteristics of the study population according to age group are summarized in Table 1; statistically significant differences between age groups had small effect sizes (Cramér’s *V*, 0.14–0.23; r, 0.18–0.23).

Successful recanalization (mTICI 2b–3) was achieved in 191 patients (82.7%): mTICI 2b in 21 (9.1%) and mTICI 3 in 170 (73.6%), whereas 40 patients (17.3%) had mTICI 0–2a. The successful recanalization rate was 85.1% (154/181) in patients aged 18–79 years and 74.0% (37/50) in those aged ≥ 80 years (*p* = 0.067). Across the three reperfusion categories, puncture-to-recanalization time (*p* = 0.004; η2H = 0.04) and baseline NIHSS score (*p* = 0.005; η2H = 0.04) differed significantly, with small effect sizes. In Bonferroni-adjusted pairwise comparisons, these differences were confined to mTICI 0–2a versus mTICI 3 (puncture-to-recanalization time, 60 vs. 45 min, adjusted *p* = 0.003; NIHSS, 16 vs. 14, adjusted *p* = 0.014), whereas mTICI 2b did not differ significantly from either mTICI 0–2a or mTICI 3 (all adjusted *p* ≥ 0.137). The small mTICI 2b group (*n* = 21) limited the power of these comparisons. The lack of a significant difference between mTICI 2b and mTICI 3 does not establish equivalence. No other baseline, procedural, or outcome variable differed significantly among the three groups (Table 2).

Among patients with successful recanalization, the rate of FR was 59.7% (114/191). Dyslipidemia (*p* = 0.022), higher admission NIHSS score (*p* < 0.001), longer puncture-to-recanalization time (*p* = 0.049), elevated NLR (*p* = 0.006), higher admission glucose level (*p* = 0.004), and higher admission serum creatinine level (*p* = 0.019) were all significantly associated with FR. Patients with FR also had significantly higher rates of ICH (*p* < 0.001) and longer hospital stays (*p* < 0.001), and all patients requiring decompressive surgery belonged to the FR group (*p* < 0.001). Baseline mRS was not significantly associated with FR (*p* = 0.143). The largest effect sizes were observed for baseline NIHSS score (r = 0.41), ASPECTS (r = 0.36), and ICH (V = 0.31), other statistically significant differences had small effect sizes (*r* or *V*, 0.14–0.29) (Table 3).

In the multivariable logistic regression Model 1 (*n* = 191), each 1-point increase in baseline NIHSS score was associated with a 29% increase in the odds of FR (OR, 1.29; 95% CI, 1.18–1.42; *p* < 0.001), each 1 mg/dL increase in admission glucose level increased the odds by 1% (OR, 1.01; 95% CI, 1.00–1.02; *p* = 0.001), and each 1-min increase in puncture-to-recanalization time increased the odds of FR by 2% (OR, 1.02; 95% CI, 1.00–1.03; *p* = 0.036). Dyslipidemia was associated with FR in univariable analysis (OR, 2.14; 95% CI, 1.11–4.13; *p* = 0.024) but did not remain in the final model. In Model 2, which included imaging variables (*n* = 146), dyslipidemia (OR, 3.51; 95% CI, 1.21–10.18; *p* = 0.021), each 1-point increase in baseline NIHSS score (OR, 1.30; 95% CI, 1.14–1.47; *p* < 0.001), each 1-unit increase in NLR (OR, 1.14; 95% CI, 1.02–1.28; *p* = 0.017), and each 1-min increase in puncture-to-recanalization time (OR, 1.02; 95% CI, 1.00–1.05; *p* = 0.019) were independently associated with higher odds of FR, whereas sufficient collateral circulation (OR, 0.19; 95% CI, 0.08–0.47; *p* < 0.001) and each 1-point increase in ASPECTS (OR, 0.60; 95% CI, 0.39–0.91; *p* = 0.017) were associated with lower odds of FR. Admission glucose was not retained in Model 2. Nagelkerke *R*^2^ was higher for Model 2 than for Model 1 (Nagelkerke *R*^2^, 0.521 vs. 0.317). Unadjusted and adjusted ORs of both models are presented in Table 4 and Figure 2.

Model 1 estimates were similar after restriction to anterior circulation infarction (*n* = 171; NIHSS: OR, 1.26 [95% CI, 1.14–1.39]; glucose: OR, 1.01 [1.00–1.02]; puncture-to-recanalization time: OR, 1.02 [1.00–1.03]). When Model 1 was refitted in the Model 2 sample (*n* = 146), NIHSS score and glucose remained significant, whereas the association of puncture-to-recanalization time was attenuated (OR, 1.01; 95% CI, 1.00–1.03; *p* = 0.105), suggesting that both imaging adjustment and sample restriction contributed to the differences between models (Supplementary Table S1). Anterior circulation patients without assessable imaging (*n* = 25) did not differ from those included in Model 2 (*n* = 146) in FR rate (64.0% vs. 60.3%; *p* = 0.724), age, sex, NIHSS score, or puncture-to-recanalization time, but had a higher NLR (median, 8.60 vs. 3.08; *p* < 0.001), a higher glucose level (*p* = 0.024) and a longer LKW-to-puncture time (*p* = 0.021) (Supplementary Table S2). After multiple imputation of the imaging variables in all 171 anterior circulation patients, the direction and magnitude of the Model 2 associations were preserved (dyslipidemia: OR, 2.80 [1.09–7.23]; NIHSS: OR, 1.21 [1.09–1.35]; sufficient collaterals: OR, 0.17 [0.07–0.40]; ASPECTS: OR, 0.61 [0.40–0.93]; puncture-to-recanalization time: OR, 1.03 [1.01–1.05]), whereas the association with NLR was attenuated and no longer statistically significant (OR, 1.09; 95% CI, 0.99–1.18; *p* = 0.065) (Supplementary Table S1).

In exploratory interaction analyses, age ≥ 80 years was not independently associated with FR when added to Model 1 (OR, 1.35; 95% CI, 0.56–3.28) or Model 2 (OR, 0.55; 95% CI, 0.16–1.81). No significant interaction was observed between age group and any predictor in Model 2 (all *p* for interaction ≥ 0.078) or between age group and NIHSS score or puncture-to-recanalization time in Model 1. A significant interaction was found between age group and admission glucose in Model 1 (*p* for interaction = 0.011): glucose was associated with FR in patients aged 18–79 years (OR, 1.02 per mg/dL; 95% CI, 1.01–1.03) but not in those aged ≥ 80 years (OR, 0.99; 95% CI, 0.98–1.01). The small subgroup aged ≥ 80 years (*n* = 37) limits interpretation of this interaction, which remains hypothesis-generating (Supplementary Table S3).

ROC analyses were performed in the Model 2 sample (*n* = 146) (Table 5 and Figure 3). Among the individual predictors, baseline NIHSS score had the highest discriminative performance (AUC, 0.78; 95% CI, 0.71–0.86; optimal cutoff, ≥15; sensitivity, 73.9%; specificity, 75.9%), followed by collateral status (AUC, 0.71; 95% CI, 0.64–0.79) and ASPECTS (AUC, 0.71; 95% CI, 0.62–0.79; optimal cutoff, ≤8), whereas NLR (AUC, 0.65) and puncture-to-recanalization time (AUC, 0.59; 95% CI, 0.49–0.68; *p* = 0.076) showed limited discrimination. The combined Model 2 achieved an AUC of 0.88 (95% CI, 0.82–0.94; sensitivity, 84.1%; specificity, 79.3%), which was significantly higher than that of NIHSS score alone (*p* = 0.003) and that of the Model 1 variables in the same patients (AUC, 0.81; *p* = 0.014). After bootstrap internal validation, the optimism-corrected AUC of Model 2 was 0.84 and the calibration slope was 0.76, indicating moderate overfitting. For comparison, Model 1 achieved an AUC of 0.79 (95% CI, 0.73–0.86) in all 191 patients. A nomogram based on Model 2, together with its calibration plot, is presented in Figure 4.

Collateral status was available for 172 patients with anterior circulation infarction in the full cohort. Insufficient collateral circulation was more frequent in patients aged ≥ 80 years than in those aged 18–79 years (81.1% [30/37] vs. 49.6% [67/135]; *p* < 0.001; *V* = 0.26), and successful recanalization was numerically less frequent in patients with insufficient than in those with sufficient collaterals (80.4% vs. 90.7%; *p* = 0.063). Among patients with successful recanalization, FR occurred in 79.5% (62/78) of patients with insufficient collaterals compared with 38.2% (26/68) of those with sufficient collaterals (*p* < 0.001; *V* = 0.42); this association was present in both age groups (18–79 years, *p* < 0.001; ≥80 years, *p* = 0.005).

ASPECTS was significantly lower in patients with FR (*p* < 0.001; r = 0.36). The association was significant in patients aged 18–79 years (*p* < 0.001), but not in the smaller subgroup aged ≥ 80 years (*n* = 28; *p* = 0.713).

Among patients with successful recanalization, those aged ≥ 80 years (*n* = 37) were more often female (70.3% vs. 44.2%; *p* = 0.004) and more frequently had hypertension (81.1% vs. 55.8%; *p* = 0.005) and AF (43.2% vs. 20.8%; *p* = 0.005) than those aged 18–79 years (*n* = 154). They also had a higher baseline NIHSS score (median, 16 vs. 14; *p* = 0.011), a different distribution of baseline mRS (*p* = 0.002), less frequent sufficient collateral circulation (17.9% vs. 53.4%; *p* < 0.001), a higher creatinine level (median, 1.00 vs. 0.89 mg/dL; *p* = 0.008), higher in-hospital mortality (48.6% vs. 27.3%; *p* = 0.012) and a longer hospital stay (median, 20 vs. 16 days; *p* = 0.021). The FR rate was numerically higher in patients aged ≥ 80 years (70.3% vs. 57.1%), but the difference was not statistically significant (*p* = 0.144). Statistically significant differences between age groups had small-to-moderate effect sizes (Cramér’s *V*, 0.18–0.28; *r*, 0.17–0.19) (Table 6). The distribution of 90-day mRS scores in patients with successful recanalization according to age group, baseline mRS, baseline NIHSS score, and mTICI category is illustrated in Figure 5.

## 4. Discussion

The present study provides several clinically relevant findings regarding FR in patients with AIS undergoing EVT. First, despite a high rate of successful angiographic recanalization, FR remained common, occurring in nearly 60% of successfully reperfused patients, a frequency consistent with previously published data [5,21,22,23]. Baseline neurological severity and procedural duration were independently associated with FR in both models. Admission glucose was retained only in Model 1, whereas collateral status, ASPECTS, dyslipidemia, and NLR were retained in the anterior circulation model incorporating imaging variables (Model 2). Hemorrhagic complications were also more frequent in patients with FR. Age ≥ 80 years was not independently associated with FR. The only significant interaction with age involved admission glucose in Model 1.

Although current European Stroke Organisation (ESO) and European Society for Minimally Invasive Neurological Therapy (ESMINT) guidelines recommend that advanced age alone should not preclude mechanical thrombectomy, the influence of age on functional recovery following EVT remains controversial [24]. Meta-analyses of randomized controlled trials have consistently shown that elderly patients derive a treatment benefit comparable to that of younger patients, yet their long-term functional outcomes remain significantly worse [5,25]. This discrepancy suggests that factors other than successful recanalization contribute substantially to outcome in older individuals. Our findings support this concept. Although patients younger than 80 years demonstrated numerically higher rates of successful recanalization, the difference was not statistically significant, indicating that the technical success of thrombectomy is largely independent of age.

Age groups also differed in their clinical and imaging characteristics. Among patients with successful recanalization, those aged ≥ 80 years had less frequent sufficient collateral circulation, higher baseline NIHSS scores, a different distribution of pre-stroke mRS, more frequent atrial fibrillation and hypertension, higher serum creatinine levels, and greater in-hospital mortality (Table 6). Cardioembolic stroke was also more frequent in this age group in the full cohort.

Lower ASPECTS was associated with poor 90-day outcome in patients aged 18–79 years, but not in those aged ≥ 80 years. Only 28 older patients had assessable baseline imaging, which limits interpretation of this subgroup result. The difference in statistical significance between age groups does not establish an age-dependent association. No significant interaction was detected between age group and ASPECTS or any other retained predictor in Model 2 (all interaction *p* values ≥ 0.078). Age ≥ 80 years was not independently associated with FR in either model (Model 1: OR, 1.35; 95% CI, 0.56–3.28; Model 2: OR, 0.55; 95% CI, 0.16–1.81). The wide confidence intervals and small subgroup size leave substantial uncertainty about age-related differences.

The differences between age groups raise the question of whether comorbidity and physiological reserve provide prognostic information beyond chronological age. This study could not directly address that question. Frailty was more prevalent among patients aged ≥ 80 years in the full cohort (Table 1), but the difference was not significant among successfully recanalized patients (Table 6), and mFI-5 was not significantly associated with FR (Table 3). The index may not capture all relevant aspects of vulnerability: three of its five components were represented elsewhere in the analysis, and it does not assess sarcopenia or cognitive reserve. Whether a more comprehensive frailty assessment improves prognostic evaluation after EVT requires further study.

Higher baseline NIHSS was the strongest clinical predictor of FR in the present study. As a well-established indicator of stroke severity, a higher NIHSS reflects more extensive neurological impairment and is often associated with larger infarct cores and poorer collateral circulation. Although patients presenting with severe neurological deficits generally derive greater benefit from EVT than from medical therapy alone, previous studies have consistently demonstrated that the likelihood of FR increases with increasing stroke severity, in agreement with our findings [23,26,27,28]. Among the individual predictors, baseline NIHSS provided the best discrimination (AUC, 0.78), with a cohort-derived cutoff of ≥15. In Model 1, each one-point increase in NIHSS increased the odds of FR by 29%. These findings support the use of baseline NIHSS for early prognostic assessment. However, the NIHSS and procedural-duration cutoffs were derived from this cohort and have not been externally validated. They should not be used as clinical decision thresholds.

The associations of metabolic and inflammatory variables with FR differed between models. In univariable analyses, patients with FR more frequently had dyslipidemia and had higher admission glucose, serum creatinine, and NLR (Table 3). Hypertension, diabetes mellitus, and atrial fibrillation, previously reported as predictors of FR [26], did not differ significantly between outcome groups. Dyslipidemia was retained in Model 2 (OR, 3.51; 95% CI, 1.21–10.18; *p* = 0.021), but not in Model 1, and the association persisted after multiple imputation (OR, 2.80; 95% CI, 1.09–7.23). Chronic endothelial dysfunction, atherosclerosis, and impaired microvascular integrity could contribute to this association by limiting tissue reperfusion after arterial reopening [29]. These mechanisms were not measured, however, and a causal interpretation is not justified. Serum creatinine was associated with FR only in univariable analysis and was not retained in either model.

Admission hyperglycemia has been associated with poor outcome after AIS and may reflect pre-existing DM or an acute stress response [30]. Previous studies have linked elevated glucose to unsuccessful recanalization, hemorrhagic transformation, and poor functional outcome after EVT [31,32]. In this cohort, glucose was independently associated with FR in Model 1 (OR, 1.01 per mg/dL; 95% CI, 1.00–1.02; *p* = 0.001), equivalent to approximately 10% higher odds per 10 mg/dL increase. Glucose was not retained in Model 2, although its univariable association remained significant in the same sample (*p* = 0.025). In Model 1, the association differed by age group (interaction *p* = 0.011): it was significant in patients aged 18–79 years (OR, 1.02 per mg/dL; 95% CI, 1.01–1.03), but not in those aged ≥ 80 years (OR, 0.99; 95% CI, 0.98–1.01). The small older subgroup warrants caution. The change between models may relate to imaging adjustment or sample restriction and does not establish that ischemic changes or collateral status mediate the glucose association. These observational findings also do not establish whether acute glucose correction reduces FR.

NLR was retained in Model 2 (OR, 1.14 per unit; 95% CI, 1.02–1.28; *p* = 0.017), but not in Model 1. After multiple imputation, the association weakened and was no longer statistically significant (OR, 1.09; 95% CI, 0.99–1.18; *p* = 0.065). Discrimination by NLR alone was limited (AUC, 0.65; 95% CI, 0.55–0.74). Its association with FR should therefore be considered exploratory. NLR is an accessible marker of systemic inflammation and has been linked to oxidative stress, endothelial injury, blood–brain barrier disruption, and microvascular thrombosis [33,34]. These processes may impair tissue reperfusion after arterial reopening, but their contribution was not directly assessed in this study.

Longer puncture-to-recanalization time was independently associated with FR in both models, with approximately 2% higher odds per additional minute (Model 1: OR, 1.02; 95% CI, 1.00–1.03; Model 2: OR, 1.02; 95% CI, 1.00–1.05). Procedural duration alone provided limited discrimination (AUC, 0.59; 95% CI, 0.49–0.68; *p* = 0.076), so the cohort-derived threshold of approximately 44 min cannot be recommended for clinical use. Longer procedures may reflect difficult vascular access, unfavorable aortic arch anatomy, or repeated thrombectomy passes. As the number of passes was not recorded, we could not distinguish the contribution of elapsed time from that of technical complexity.

Collateral status was strongly associated with FR. Sufficient collateral circulation was associated with lower odds of FR in Model 2 (OR, 0.19; 95% CI, 0.08–0.47; *p* < 0.001), and the association persisted after multiple imputation (OR, 0.17; 95% CI, 0.07–0.40). The univariable association was present in both age groups. Collateral flow sustains perfusion of the ischemic penumbra before recanalization and limits infarct growth [15,22]. Poor collateral supply may therefore allow irreversible injury to develop before arterial reopening, reducing the likelihood of functional recovery despite successful recanalization.

Hemorrhagic complications were more frequent in patients with FR: any intracranial hemorrhage occurred in 36.8% versus 9.1%, and symptomatic intracranial hemorrhage in 17.5% versus 2.6%. All 19 patients requiring decompressive surgery had FR. Hemorrhagic transformation after EVT involves ischemic blood–brain barrier disruption, reperfusion injury, and inflammation [8,9]. These processes may overlap with the mechanisms underlying FR. Because hemorrhage occurs after reperfusion and may contribute to subsequent disability, it was not included in the prognostic regression models. The observed association does not establish its independent causal contribution to FR.

Previous studies have suggested that bridging therapy may reduce the likelihood of FR [5]. Bridging therapy was not significantly associated with FR in our cohort (Table 3), and its frequency did not differ between age groups among patients with successful recanalization (Table 6). It did not meet the entry criterion for either multivariable model. Approximately 47% of successfully recanalized patients received intravenous thrombolysis before EVT; contraindications prevented its use in many patients. Comparisons of eligible and ineligible patients are susceptible to treatment-selection bias and cannot establish a treatment effect. The absence of an association in this study does not alter guideline recommendations to administer IV rtPA before EVT in eligible patients [2].

The benefit of EVT in posterior circulation stroke has been demonstrated by the BAOCHE [35] and ATTENTION [36] trials, both of which reported superior functional outcomes compared with best medical therapy alone. Nevertheless, basilar artery occlusion remains technically more challenging and is frequently associated with longer procedural times, lower recanalization rates, and a higher risk of FR than anterior circulation stroke [28]. In our cohort, all 25 patients with posterior circulation stroke had basilar artery occlusion. Successful recanalization was achieved in 20 patients, while FR occurred in 50% of successfully treated cases. Because of the relatively small number of posterior circulation strokes included in our study, these findings should be interpreted with caution and require confirmation in larger patient cohorts.

The present study has several strengths. It included a consecutive cohort treated according to a standardized EVT protocol, with recanalization and FR rates consistent with those reported previously, and it allowed clinical, laboratory, imaging, and procedural determinants of FR to be evaluated together. Age interactions were formally tested, and Model 2 underwent bootstrap internal validation. Sensitivity analyses and multiple imputation supported the principal findings, although the association with NLR was attenuated after imputation.

The retrospective, single-center design leaves the results susceptible to selection bias and residual confounding. Few patients had posterior circulation stroke, limiting subgroup analyses. Model 2 was restricted to 146 anterior circulation patients with assessable baseline imaging, and the primary analyses were therefore complete-case analyses. Sensitivity analyses and multiple imputation addressed this restriction, but differences between patients with and without imaging indicate that residual bias remains possible. Age-stratified analyses were exploratory: only 50 patients in the full cohort were aged ≥ 80 years, and 37 achieved successful recanalization. The study had limited power to detect interactions, and no multiplicity adjustment was applied to these analyses. Thrombectomy pass counts were unavailable, preventing assessment of the first-pass effect and adjustment for procedural complexity. Grade mTICI 2c was not recorded separately, and the small mTICI 2b group limited comparisons with mTICI 3. The absence of a significant difference between these categories does not establish equivalence, and the difference in FR between mTICI 2b and mTICI 3 requires confirmation in larger cohorts. ROC cutoffs and the nomogram were developed in the same dataset, and Model 2 showed moderate overfitting on internal validation; external validation is required before clinical use. Finally, all patients were functionally independent before stroke. Dichotomizing the 90-day mRS does not capture change from pre-stroke function, and the findings may not apply to patients with pre-existing disability.

## 5. Conclusions

FR remains a major clinical challenge despite technically successful EVT for AIS. The present study demonstrates that functional recovery following EVT depends on substantially more than successful angiographic reperfusion alone. Baseline neurological severity and puncture-to-recanalization time were independently associated with FR in both models. Admission glucose was retained only in Model 1. Collateral status, ASPECTS, dyslipidemia, and NLR were retained in Model 2, which included imaging variables in patients with anterior circulation infarction. The NLR association weakened after multiple imputation and remains exploratory.

Baseline NIHSS, ASPECTS, and collateral status may assist prognostic assessment and patient counseling. Procedural duration is potentially modifiable, but its association with FR may also reflect technical complexity. Model 2 showed good apparent discrimination, with some loss of performance on internal validation; the model and its thresholds require external validation before clinical application. Age ≥ 80 years was not independently associated with FR, and the glucose interaction in Model 1 was the only significant age interaction. These exploratory findings require confirmation in larger cohorts with sufficient power to evaluate effect modification.

Future large-scale prospective multicenter studies are warranted to validate these findings and to develop robust prediction models that may ultimately enhance the clinical benefit of EVT.

## Figures and Tables

**Figure 1:**
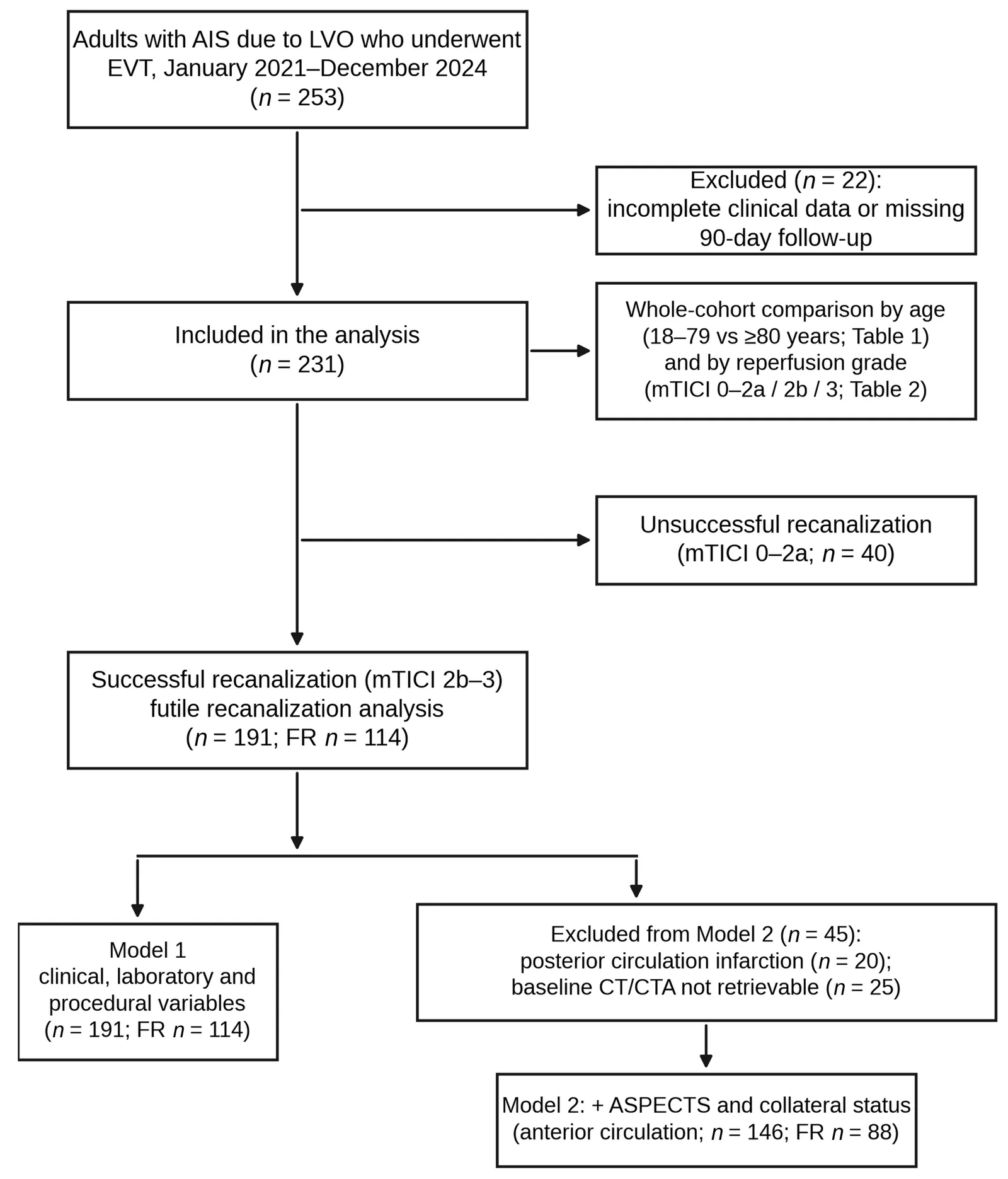
Flow diagram of patient selection and of the samples used in each analysis. AIS, acute ischemic stroke; ASPECTS, Alberta Stroke Program Early CT Score; CTA, computed tomography angiography; EVT, endovascular therapy; FR, futile recanalization; LVO, large vessel occlusion; mTICI, modified Thrombolysis in Cerebral Infarction.

**Figure 2:**
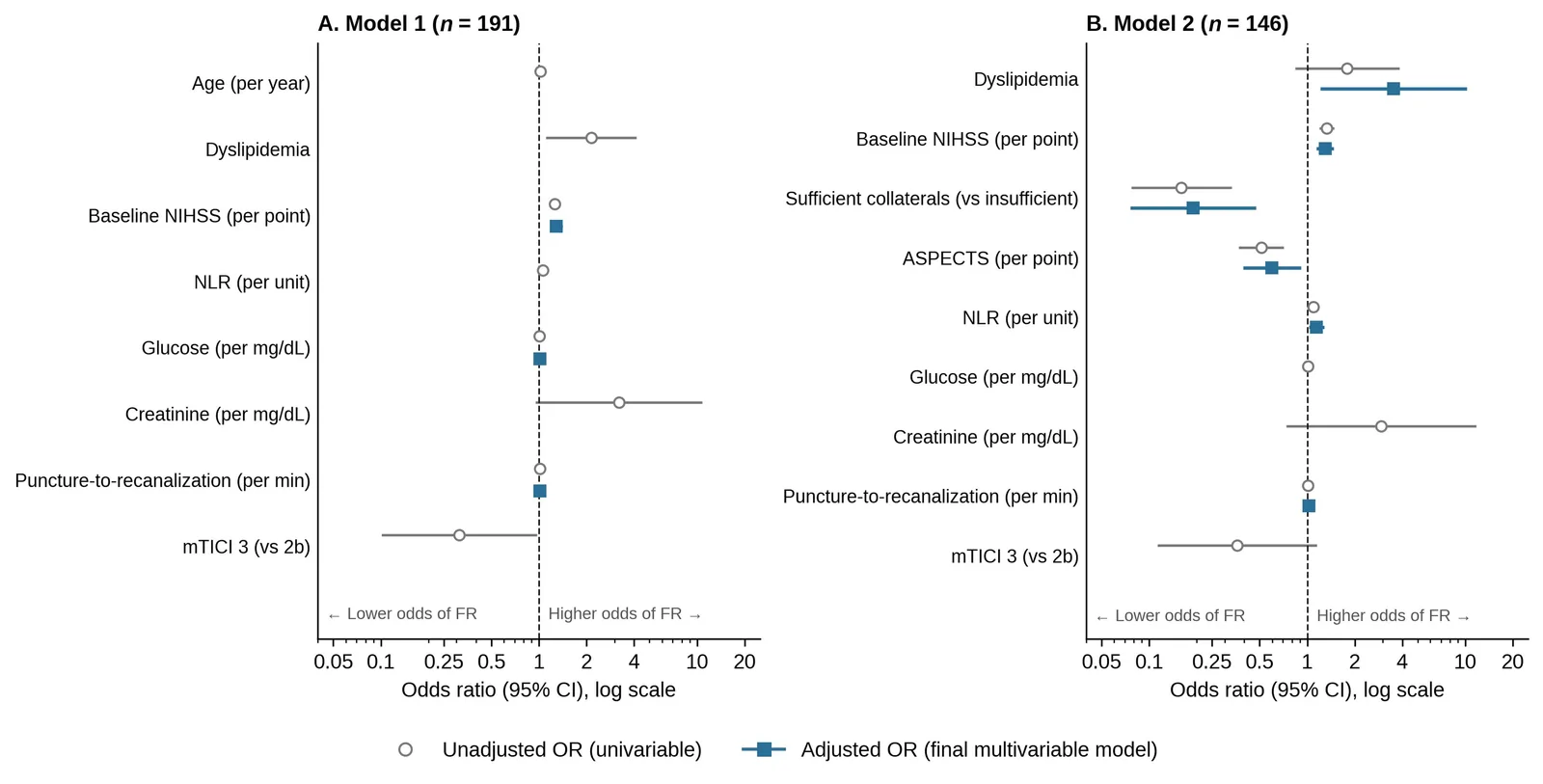
Forest plot of unadjusted (open circles; univariable logistic regression) and adjusted (filled squares; final multivariable model) odds ratios with 95% confidence intervals for futile recanalization (FR) in Model 1 ((**A**); *n* = 191) and Model 2 ((**B**); *n* = 146). Adjusted estimates are shown only for variables retained in the final models after backward Wald elimination; all candidate variables with *p* < 0.20 in univariable analysis are displayed. The x-axis is logarithmic. ASPECTS, Alberta Stroke Program Early CT Score; mTICI, modified Thrombolysis in Cerebral Infarction; NIHSS, National Institutes of Health Stroke Scale; NLR, neutrophil-to-lymphocyte ratio.

**Figure 3:**
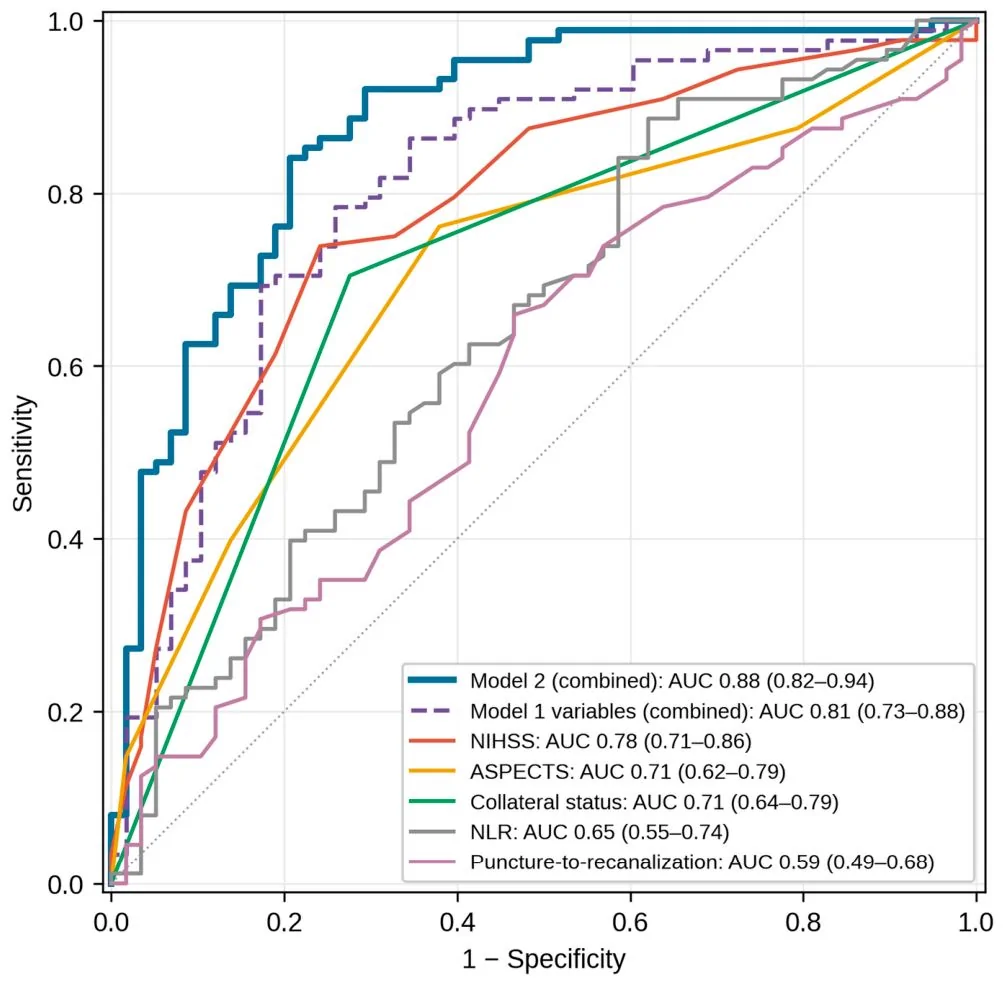
Receiver operating characteristic (ROC) curves of the combined Model 2, the combined Model 1 variables, and the individual predictors retained in Model 2 for the discrimination of futile recanalization in the Model 2 sample (*n* = 146). Areas under the curve (AUC) with 95% confidence intervals (DeLong method) are given in the legend. The dashed diagonal line indicates the line of no discrimination (AUC = 0.50). Optimal cutoff values, sensitivity, and specificity are presented in Table 5. ASPECTS, Alberta Stroke Program Early CT Score; NIHSS, National Institutes of Health Stroke Scale; NLR, neutrophil-to-lymphocyte ratio.

**Figure 4:**
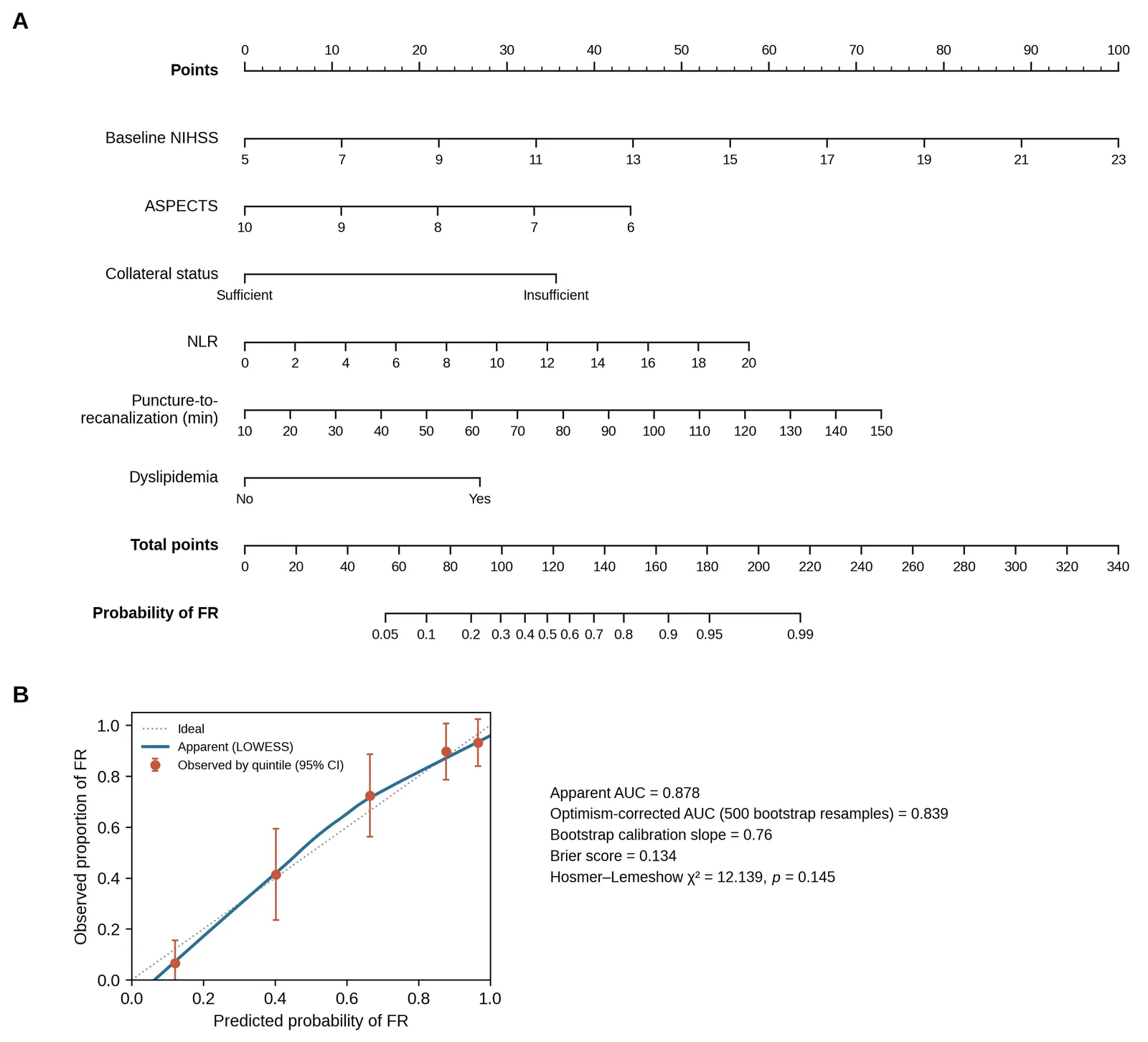
(**A**) Nomogram based on Model 2 for estimating the individual probability of futile recanalization (FR) after successful reperfusion (mTICI 2b–3) in patients with anterior circulation infarction. For each predictor, the patient’s value is located on its axis and a vertical line is drawn up to the Points axis; the points of all predictors are summed, and the total is projected vertically from the Total points axis onto the Probability of FR axis. For example, a patient with dyslipidemia, NIHSS score of 16, insufficient collateral status, ASPECTS of 8, NLR of 4, and a puncture-to-recanalization time of 45 min receives approximately 175 points, corresponding to a predicted FR probability of approximately 94%. The NLR axis is truncated at 20 (approximately the 97.5th percentile). (**B**) Calibration of Model 2: apparent LOWESS-smoothed calibration curve and observed proportions of FR (with 95% confidence intervals) by quintile of predicted probability; the dotted line indicates perfect calibration. The optimism-corrected area under the curve (AUC) and calibration slope were obtained with 500 bootstrap resamples in which the full variable selection procedure was repeated. ASPECTS, Alberta Stroke Program Early CT Score; NIHSS, National Institutes of Health Stroke Scale; NLR, neutrophil-to-lymphocyte ratio.

**Figure 5:**
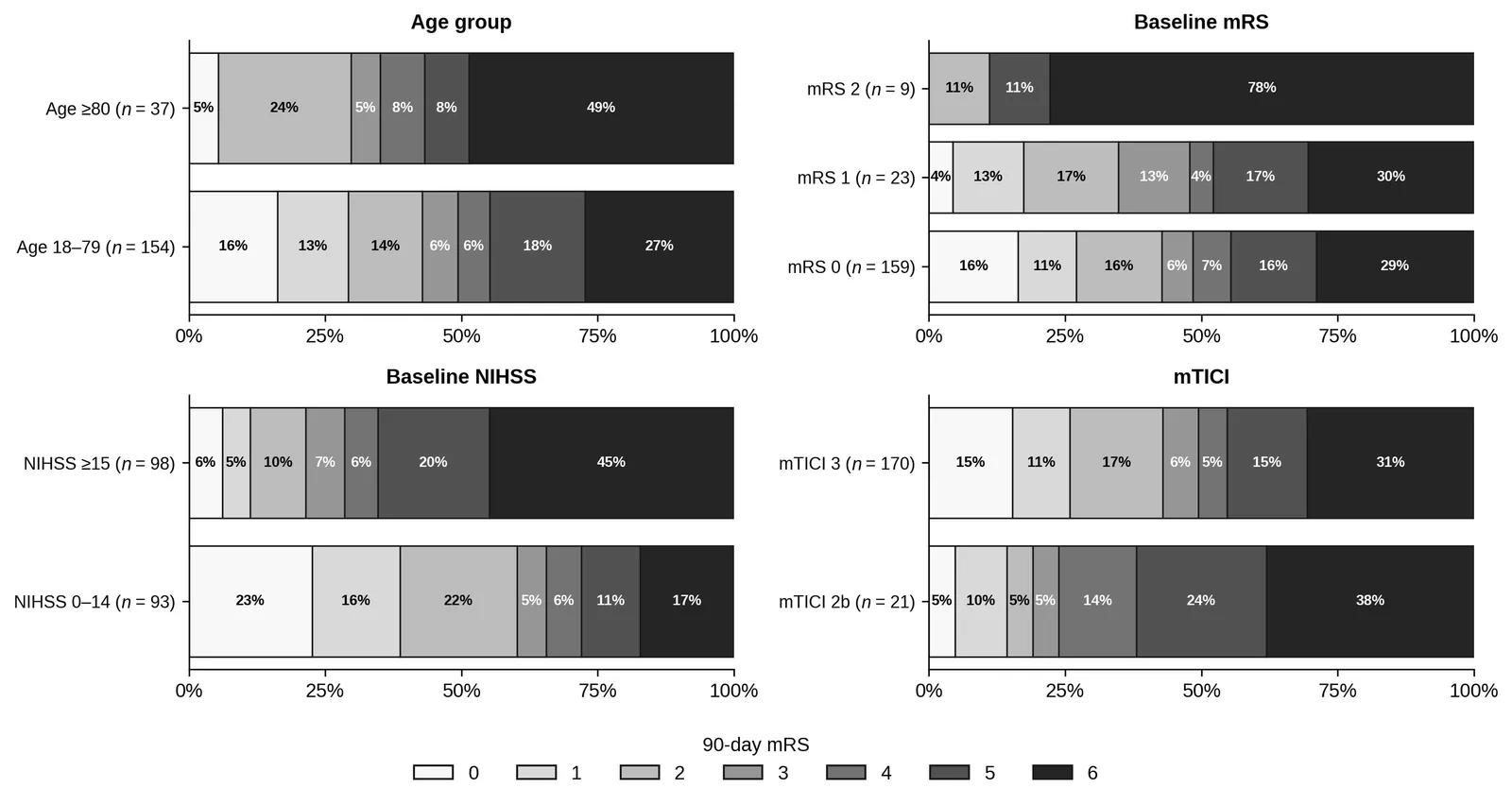
Distribution of 90-day modified Rankin Scale scores according to age group, baseline mRS, baseline stroke severity, and reperfusion grade in patients with successful recanalization (*n* = 191). Stacked bars show the distribution of modified Rankin Scale (mRS) scores at 90 days within each subgroup. Numbers within the bars denote patient percent, whereas segment widths represent the proportion of the subgroup, as indicated on the x-axis. Subgroups were defined by age (18–79 vs. ≥80 years), baseline mRS (mRS 0, mRS 1, and mRS 2), baseline stroke severity (NIHSS 0–14 vs. ≥15), and final angiographic reperfusion grade (mTICI 2b vs. 3). Segments are absent where no patient had the corresponding mRS score; percentages < 3.5% are not labeled. mRS, modified Rankin Scale; mTICI, modified Thrombolysis in Cerebral Infarction; NIHSS, National Institutes of Health Stroke Scale.

**Table 1:** Baseline demographic, clinical, radiological, and laboratory characteristics of patients by age group.

	All Patients (*n* = 231)	Age 18–79 Years (*n* = 181)	Age ≥ 80 Years (*n* = 50)	*p* Value	Effect Size ^g^
**Demographics and comorbidities**					
Age, median [IQR]	71 [16]	66 [17]	83 [5]	—	—
Sex, male, *n* (%)	118 (51.1)	102 (56.4)	16 (32.0)	**0.002** ^a^	0.20
Frailty, *n* (%) *	77 (33.3)	54 (29.8)	23 (46.0)	**0.032** ^a^	0.14
Hypertension, *n* (%)	142 (61.5)	101 (55.8)	41 (82.0)	**0.001** ^a^	0.22
Diabetes mellitus, *n* (%)	65 (28.1)	49 (27.1)	16 (32.0)	0.493 ^a^	0.05
Atrial fibrillation, *n* (%)	61 (26.4)	38 (21.0)	23 (46.0)	**<0.001** ^a^	0.23
Dyslipidemia, *n* (%)	75 (32.5)	61 (33.7)	14 (28.0)	0.446 ^a^	0.05
Chronic kidney disease, *n* (%)	7 (3.0)	3 (1.7)	4 (8.0)	**0.041** ^a^	0.15
Prior antiplatelet medication, *n* (%)	40 (17.3)	30 (16.6)	10 (20.0)	0.571 ^a^	0.04
Prior anticoagulant medication, *n* (%)	42 (18.2)	25 (13.8)	17 (34.0)	**0.001** ^a^	0.22
**Baseline severity scores**					
NIHSS score at admission, median [IQR]	15 [6]	14 [6]	16 [4]	**0.006** ^b^	0.18
ASPECTS (*n* = 172), median [IQR] **	8 [2]	8 [2]	7 [1]	**0.010** ^b^	0.20
**Laboratory data**					
NLR, median [IQR]	3.40 [5.36]	3.33 [5.28]	3.95 [6.16]	0.846 ^b^	0.08
Glucose, mg/dL, median [IQR]	131 [53]	131 [49]	132 [62]	0.621 ^b^	0.03
Creatinine, mg/dL, median [IQR]	0.90 [0.26]	0.89 [0.22]	1.01 [0.37]	**0.010** ^b^	0.23
**Vascular territory**				0.215 ^a^	0.08
Anterior circulation infarct, *n* (%)	206 (89.2)	159 (87.8)	47 (94.0)		
Posterior circulation infarct, *n* (%)	25 (10.8)	22 (12.2)	3 (6.0)		
**Outcome measures**					
In-hospital mortality, *n* (%)	79 (34.2)	52 (28.7)	27 (54.0)	**<0.001** ^a^	0.22

Data are presented as *n* (%) for categorical variables and median [interquartile range, IQR] for continuous variables. ^a^ Pearson chi-square test or Fisher’s exact test (expected cell count < 5); ^b^ Mann–Whitney U test. *p* values refer to the comparison between the 18–79-year and ≥80-year subgroups. Bold *p* values indicate statistical significance (*p* < 0.05). ^g^ Effect size: Cramér’s *V* (φ) for categorical variables and *r* = |*Z*|/√*N* for continuous variables (Mann–Whitney U test); values of approximately 0.10, 0.30 and 0.50 indicate small, medium, and large effects, respectively. * Frailty was assessed using the 5-factor modified Frailty Index (mFI-5). ** ASPECTS was calculated only in patients with anterior circulation infarction and 34 patients were excluded because their images could not be accessed for review (*n* = 172). ASPECTS, Alberta Stroke Program Early CT Score; IQR, interquartile range; NIHSS, National Institutes of Health Stroke Scale; NLR, neutrophil-to-lymphocyte ratio.

**Table 2:** Baseline characteristics, procedural details, and clinical outcomes according to mTICI 0–2a, mTICI 2b, and mTICI 3 recanalization.

	mTICI 0–2a (*n* = 40)	mTICI 2b (*n* = 21)	mTICI 3 (*n* = 170)	*p* Value	Pairwise Adjusted *p* ^f^	Effect Size ^g^
**Demographics and comorbidities**						
Age ≥ 80 years, *n* (%)	13 (32.5)	6 (28.6)	31 (18.2)	0.095 ^a^		0.14
Sex, male, *n* (%)	21 (52.5)	10 (47.6)	87 (51.2)	0.935 ^a^		0.02
Frailty, *n* (%) *	18 (45.0)	8 (38.1)	51 (30.0)	0.173 ^a^		0.12
Hypertension, *n* (%)	26 (65.0)	12 (57.1)	104 (61.2)	0.826 ^a^		0.04
Diabetes mellitus, *n* (%)	16 (40.0)	5 (23.8)	44 (25.9)	0.182 ^a^		0.12
Atrial fibrillation, *n* (%)	13 (32.5)	4 (19.0)	44 (25.9)	0.503 ^a^		0.08
Dyslipidemia, *n* (%)	15 (37.5)	5 (23.8)	55 (32.4)	0.554 ^a^		0.07
Chronic kidney disease, *n* (%)	3 (7.5)	1 (4.8)	3 (1.8)	0.088 ^a^		0.13
**Procedural characteristics**						
Last known well-to-groin puncture time, min, median [IQR]	322 [172]	314 [147]	288 [175]	0.876 ^e^		<0.01
Groin puncture-to-recanalization time, min, median [IQR]	60 [47]	50 [16]	45 [20]	**0.004** ^e^	0–2a vs. 2b: 0.613; **0–2a vs. 3: 0.003**; 2b vs. 3: 0.911	0.04
Bridging therapy (IV thrombolysis), *n* (%)	18 (45.0)	12 (57.1)	78 (45.9)	0.603 ^a^		0.07
**Baseline severity scores**						
NIHSS score, median [IQR]	16 [7]	17 [6]	14 [6]	**0.005** ^e^	0–2a vs. 2b: >0.999; **0–2a vs. 3: 0.014**; 2b vs. 3: 0.137	0.04
ASPECTS (*n* = 172), median [IQR] **	8 [2]	7 [3]	8 [2]	0.168 ^e^		0.01
**Laboratory data**						
Glucose, mg/dL, median [IQR]	133 [57]	142 [63]	131 [46]	0.703 ^e^		<0.01
Creatinine, mg/dL, median [IQR]	0.9 [0.23]	0.91 [0.29]	0.90 [0.25]	0.691 ^e^		<0.01
**Outcome measures**						
Length of hospital stay, days, median [IQR]	25 [41]	20 [25]	16 [18]	0.118 ^e^		0.01
In-hospital mortality, *n* (%)	19 (47.5)	8 (38.1)	52 (30.6)	0.118 ^a^		0.14
**Complications**						
Intracranial hemorrhage, *n* (%)	12 (30.0)	7 (33.3)	42 (24.7)	0.595 ^a^		0.07
Symptomatic intracranial hemorrhage, *n* (%)	6 (15.0)	1 (4.8)	21 (12.4)	0.556 ^a^		0.08

Data are presented as *n* (%) for categorical variables and median [interquartile range, IQR] for continuous variables. ^a^ Pearson chi-square test or Fisher’s exact test (expected cell count < 5); ^e^ Kruskal-Wallis test; ^f^ Post hoc pairwise comparisons with Dunn’s test and Bonferroni adjustment, performed only when the overall Kruskal–Wallis test was significant; significant pairs are shown in bold. ^g^ Effect size: Cramér’s *V* for categorical variables and η2H = (*H* − *k* + 1)/(*n* − *k*) for continuous variables (Kruskal–Wallis test); for η2H, values of 0.01, 0.06 and 0.14 indicate small, medium, and large effects, respectively. mTICI grade 2c was not separately recorded in the study database; mTICI 3 denotes complete reperfusion. Bold *p* values indicate statistical significance (*p* < 0.05). * Frailty was assessed using the 5-factor modified Frailty Index (mFI-5). ** ASPECTS was calculated only in patients with anterior circulation infarction and which the imaging measures were applicable (*n* = 172). ASPECTS, Alberta Stroke Program Early CT Score; IQR, interquartile range; IV, intravenous; mTICI, modified Thrombolysis in Cerebral Infarction; NIHSS, National Institutes of Health Stroke Scale.

**Table 3:** Baseline characteristics, procedural details, and complications according to mRS > 2 at 90 days versus mRS 0–2 among patients with successful recanalization (mTICI 2b–3).

	mRS > 2 (*n* = 114)	mRS 0–2 (*n* = 77)	*p* Value	Effect Size ^g^
**Demographics and comorbidities**				
Age ≥ 80 years, *n* (%)	26 (22.8)	11 (14.3)	0.144 ^a^	V = 0.11
Sex, male, *n* (%)	59 (51.8)	38 (49.4)	0.744 ^a^	V = 0.02
Hypertension, *n* (%)	72 (63.2)	44 (57.1)	0.404 ^a^	V = 0.06
Diabetes mellitus, *n* (%)	28 (24.6)	21 (27.3)	0.674 ^a^	V = 0.03
Atrial fibrillation, *n* (%)	29 (25.4)	19 (24.7)	0.905 ^a^	V = 0.01
Dyslipidemia, *n* (%)	43 (37.7)	17 (22.1)	**0.022** ^a^	V = 0.17
Chronic kidney disease, *n* (%)	4 (3.5)	0 (0.0)	0.149 ^a^	V = 0.12
Baseline mRS			0.143 ^a^	V = 0.14
mRS 0, *n* (%)	91 (79.8)	68 (88.3)		
mRS 1, *n* (%)	15 (13.2)	8 (10.4)		
mRS 2, *n* (%)	8 (7)	1 (1.3)		
Frailty, *n* (%) *	36 (31.6)	23 (29.9)	0.802 ^a^	V = 0.02
**Baseline severity scores**				
NIHSS score, median [IQR]	16 [5]	12 [6]	**<0.001** ^b^	r = 0.41
ASPECTS (*n* = 146), median [IQR] **	8 [1]	9 [1]	**<0.001** ^b^	r = 0.36
**Laboratory data**				
NLR, median [IQR]	3.96 [6.2]	3.03 [4.51]	**0.006** ^b^	r = 0.20
Glucose, mg/dL, median [IQR]	138 [63]	119 [34]	**0.004** ^b^	r = 0.21
Creatinine, mg/dL, median [IQR]	0.93 [0.31]	0.85 [0.17]	**0.019** ^b^	r = 0.17
**Procedural characteristics**				
Anterior circulation infarct, *n* (%)	104 (91.2)	67 (87.0)	0.351 ^a^	V = 0.07
Posterior circulation infarct, *n* (%)	10 (8.8)	10 (13.0)	0.351 ^a^	V = 0.07
LKW-to-puncture time, min, median [IQR]	303.5 [156]	273 [166]	0.181 ^b^	r = 0.10
Puncture-to-recanalization time, min, median [IQR]	48 [29]	42 [27]	**0.049** ^b^	r = 0.14
Bridging therapy (IV thrombolysis), *n* (%)	54 (47.4)	36 (46.8)	0.933 ^a^	V = 0.01
LKW-to-needle time (*n* = 90), min, median [IQR]	182 [96]	155 [70]	0.067 ^b^	r = 0.19
mTICI 2b, *n* (%)	17 (14.9)	4 (5.2)	**0.035 ^a^**	V = 0.15
mTICI 3, *n* (%)	97 (85.1)	73 (94.8)		
**Outcome measures**				
Length of hospital stay, days, median [IQR]	20 [23]	15 [6]	**<0.001** ^b^	r = 0.29
**Complications**				
Intracranial hemorrhage, *n* (%)	42 (36.8)	7 (9.1)	**<0.001** ^a^	V = 0.31
Symptomatic intracranial hemorrhage, *n* (%)	20 (17.5)	2 (2.6)	**0.002** ^a^	V = 0.23
Decompressive surgery, *n* (%)	19 (16.7)	0 (0.0)	**<0.001** ^a^	V = 0.27

Futile recanalization was defined as a modified Rankin Scale (mRS) score > 2 at 90 days despite successful angiographic reperfusion (mTICI 2b–3). Data are presented as *n* (%) for categorical variables and median [interquartile range, IQR] for continuous variables. ^a^ Pearson chi-square test or Fisher’s exact test (expected cell count < 5); ^b^ Mann–Whitney U test. Bold *p* values indicate statistical significance (*p* < 0.05). ^g^ Effect size: Cramér’s *V* (φ) for categorical variables and *r* = |*Z*|/√*N* for continuous variables; values of approximately 0.10, 0.30 and 0.50 indicate small, medium, and large effects, respectively. * Frailty was assessed using the 5-factor modified Frailty Index (mFI-5). ** ASPECTS was calculated only in patients with anterior circulation infarction which the imaging measures were applicable (*n* = 146). LKW-to-needle time applies only to patients who received IV thrombolysis (*n* = 90). mTICI grade 2c was not separately recorded; mTICI 3 denotes complete reperfusion. ASPECTS, Alberta Stroke Program Early CT Score; IQR, interquartile range; IV, intravenous; LKW, last known well; mRS, modified Rankin Scale; mTICI, modified Thrombolysis in Cerebral Infarction; NIHSS, National Institutes of Health Stroke Scale; NLR, neutrophil-to-lymphocyte ratio.

**Table 4:** Univariable and multivariable logistic regression analysis of factors associated with futile recanalization after successful reperfusion (mTICI 2b–3).

Model 1. Clinical, laboratory and procedural variables (*n* = 191)	**Unadjusted OR (95% CI)**	***p** * **value**	**Adjusted OR (95% CI)**	***p** * **value**
Age (per 1-year increase)	1.02 (1.00–1.04)	0.081 ^c^	—	—
Dyslipidemia	2.14 (1.11–4.13)	**0.024** ^c^	—	—
Baseline NIHSS score (per 1-point increase)	1.26 (1.16–1.38)	**<0.001** ^c^	1.29 (1.18–1.42)	**<0.001** ^d^
NLR (per 1-point increase)	1.05 (0.99–1.12)	0.089 ^c^	—	—
Baseline glucose (per 1 mg/dL increase)	1.01 (1.00–1.02)	**0.004** ^c^	1.01 (1.00–1.02)	**0.001** ^d^
Baseline creatinine (per 1 mg/dL increase)	3.20 (0.95–10.81)	0.061 ^c^	—	—
Puncture-to-recanalization time (per 1-min increase)	1.01 (1.00–1.03)	0.059 ^c^	1.02 (1.00–1.03)	**0.036** ^d^
LKW-to-needle time (per 1-min increase; *n* = 90) ^e^	1.01 (1.00–1.02)	0.059 ^c^	—	—
mTICI 3 (versus mTICI 2b)	0.32 (0.10–0.97)	**0.044** ^c^	—	—
Model 2. Model 1 variables plus imaging variables (*n* = 146) **	**Unadjusted OR (95% CI)**	***p** * **value**	**Adjusted OR (95% CI)**	***p** * **value**
Age (per 1-year increase)	1.01 (0.99–1.04)	0.279 ^c^	—	—
Dyslipidemia	1.79 (0.84–3.82)	0.132 ^c^	3.51 (1.21–10.18)	**0.021** ^d^
Baseline NIHSS score (per 1-point increase)	1.33 (1.19–1.48)	**<0.001** ^c^	1.30 (1.14–1.47)	**<0.001** ^d^
Sufficient collateral status (vs. Insufficient collateral status)	0.16 (0.08–0.33)	**<0.001** ^c^	0.19 (0.08–0.47)	**<0.001** ^d^
ASPECTS (per 1-point increase)	0.51 (0.37–0.71)	**<0.001** ^c^	0.60 (0.39–0.91)	**0.017** ^d^
NLR (per 1-point increase)	1.09 (0.99–1.20)	0.058 ^c^	1.14 (1.02–1.28)	**0.017** ^d^
Baseline glucose (per 1 mg/dL increase)	1.01 (1.00–1.02)	**0.025** ^c^	—	—
Baseline creatinine (per 1 mg/dL increase)	2.93 (0.74–11.69)	0.127 ^c^	—	—
Puncture-to-recanalization time (per 1-min increase)	1.01 (0.99–1.03)	0.138 ^c^	1.02 (1.00–1.05)	**0.019** ^d^
LKW-to-needle time (per 1-min increase; *n* = 67) ^e^	1.01 (1.00–1.02)	0.008 ^c^	—	—
mTICI 3 (versus mTICI 2b)	0.36 (0.11–1.15)	0.084 ^c^	—	—

Futile recanalization was defined as a modified Rankin Scale score > 2 at 90 days despite successful angiographic reperfusion (mTICI 2b–3). ^c^ Odds ratios (OR) obtained from univariable logistic regression. ^d^ Within each model sample, variables with *p* < 0.20 in univariable analysis were entered simultaneously into the multivariable model and eliminated with the backward Wald method (removal at *p* > 0.10): Model 1, age, dyslipidemia, baseline NIHSS score, NLR, baseline glucose, baseline creatinine, puncture-to-recanalization time, and mTICI grade; Model 2, dyslipidemia, baseline NIHSS score, collateral status, ASPECTS, NLR, baseline glucose, baseline creatinine, puncture-to-recanalization time, and mTICI grade. ^e^ LKW-to-needle time is defined only for patients who received IV thrombolysis; its univariable OR was estimated in these patients only, and it was not entered into the multivariable models. Model fit was acceptable (Model 1: Hosmer–Lemeshow χ^2^ = 3.886, *p* = 0.876; Nagelkerke *R*^2^ = 0.317 and Model 2: Hosmer–Lemeshow χ^2^ = 12.139, *p* = 0.145; Nagelkerke *R*^2^ = 0.521); the overall model was statistically significant (omnibus test, *p* < 0.001). All variance inflation factors were <5. ** ASPECTS was calculated only in patients with anterior circulation infarction which the imaging measures were applicable (*n* = 146). Variables not retained in the final model are indicated by a dash. mTICI grade 2c was not separately recorded; mTICI 3 denotes complete reperfusion. Adjusted ORs of both models are displayed graphically in Figure 2. CI, confidence interval; LKW, last known well; mTICI, modified Thrombolysis in Cerebral Infarction; NIHSS, National Institutes of Health Stroke Scale; NLR, neutrophil-to-lymphocyte ratio; OR, odds ratio.

**Table 5:** Discriminative performance of Model 2, the combined Model 1 variables, and the individual predictors retained in Model 2 for futile recanalization (*n* = 146).

	AUC (95% CI)	*p* Value	Optimal Cutoff	Sensitivity, %	Specificity, %
Model 2, combined (predicted probability)	0.878 (0.819–0.937)	**<0.001**	≥0.56	84.1	79.3
Model 1 variables, combined (predicted probability)	0.808 (0.733–0.882)	**<0.001**	≥0.57	78.4	74.1
Baseline NIHSS score	0.783 (0.706–0.859)	**<0.001**	≥15	73.9	75.9
ASPECTS	0.707 (0.624–0.789)	**<0.001**	≤8	76.1	62.1
Insufficient collateral status	0.714 (0.639–0.790)	**<0.001**	Insufficient	70.5	72.4
NLR	0.645 (0.552–0.738)	**0.002**	≥1.89	88.6	37.9
Puncture-to-recanalization time, min	0.586 (0.491–0.681)	0.076	≥44	65.9	53.4
Dyslipidemia	0.558 (0.485–0.632)	0.120	Present	34.1	77.6

ROC analyses were performed in the Model 2 sample (patients with anterior circulation infarction and assessable imaging; *n* = 146; futile recanalization, *n* = 88). AUCs with 95% CIs and *p* values (vs. AUC = 0.5) were obtained with DeLong’s method; optimal cutoffs were determined with the Youden index. The AUC of Model 2 was significantly higher than that of the NIHSS score alone (*p* = 0.003) and that of the combined Model 1 variables in the same patients (*p* = 0.014) (DeLong test). Internal validation of Model 2 with 500 bootstrap resamples (repeating the backward selection in each resample) yielded an optimism-corrected AUC of 0.839 and a calibration slope of 0.76; Brier score, 0.134. In all 191 patients, Model 1 had an AUC of 0.794 (95% CI, 0.729–0.859). ASPECTS, Alberta Stroke Program Early CT Score; AUC, area under the curve; CI, confidence interval; NIHSS, National Institutes of Health Stroke Scale; NLR, neutrophil-to-lymphocyte ratio; ROC, receiver operating characteristic.

**Table 6:** Baseline characteristics, procedural details, and outcomes according to age group among patients with successful recanalization (mTICI 2b–3; *n* = 191).

	Age 18–79 Years (*n* = 154)	Age ≥ 80 Years (*n* = 37)	*p* Value	Effect Size ^g^
**Demographics and comorbidities**				
Sex, male, *n* (%)	86 (55.8)	11 (29.7)	**0.004** ^a^	V = 0.21
Hypertension, *n* (%)	86 (55.8)	30 (81.1)	**0.005** ^a^	V = 0.20
Diabetes mellitus, *n* (%)	39 (25.3)	10 (27.0)	0.831 ^a^	V = 0.02
Coronary artery disease, *n* (%)	43 (27.9)	5 (13.5)	0.070 ^a^	V = 0.13
Atrial fibrillation, *n* (%)	32 (20.8)	16 (43.2)	**0.005** ^a^	V = 0.20
Dyslipidemia, *n* (%)	51 (33.1)	9 (24.3)	0.301 ^a^	V = 0.07
Frailty, *n* (%) *	44 (28.6)	15 (40.5)	0.157 ^a^	V = 0.10
Baseline mRS			**0.002** ^a^	V = 0.26
mRS 0, *n* (%)	135 (87.7)	24 (64.9)		
mRS 1, *n* (%)	15 (9.7)	8 (21.6)		
mRS 2, *n* (%)	4 (2.6)	5 (13.5)		
**Baseline severity and imaging**				
NIHSS score, median [IQR]	14 [6]	16 [4]	**0.011** ^b^	r = 0.18
ASPECTS (*n* = 146), median [IQR] **	8 [2]	8 [2]	0.107 ^b^	r = 0.13
Sufficient collateral status (*n* = 146), *n* (%) **	63 (53.4)	5 (17.9)	**<0.001** ^a^	V = 0.28
Anterior circulation infarct, *n* (%)	137 (89.0)	34 (91.9)	0.770 ^a^	V = 0.04
**Laboratory data**				
NLR, median [IQR]	3.42 [5.63]	3.95 [6.38]	0.429 ^b^	r = 0.06
Glucose, mg/dL, median [IQR]	131 [46]	132 [60]	0.870 ^b^	r = 0.01
Creatinine, mg/dL, median [IQR]	0.89 [0.23]	1.00 [0.36]	**0.008** ^b^	r = 0.19
**Procedural characteristics**				
LKW-to-puncture time, min, median [IQR]	286 [178]	302 [165]	0.444 ^b^	r = 0.06
Puncture-to-recanalization time, min, median [IQR]	47 [29]	43 [22]	0.224 ^b^	r = 0.09
Bridging therapy (IV thrombolysis), *n* (%)	74 (48.1)	16 (43.2)	0.599 ^a^	V = 0.04
LKW-to-needle time (*n* = 90), min, median [IQR]	175 [90]	188 [140]	0.657 ^b^	r = 0.05
mTICI 3, *n* (%)	139 (90.3)	31 (83.8)	0.252 ^a^	V = 0.08
**Outcome measures**				
Futile recanalization (90-day mRS > 2), *n* (%)	88 (57.1)	26 (70.3)	0.144 ^a^	V = 0.11
In-hospital mortality, *n* (%)	42 (27.3)	18 (48.6)	**0.012** ^a^	V = 0.18
Length of hospital stay, days, median [IQR]	16 [16]	20 [26]	**0.021** ^b^	r = 0.17
Intracranial hemorrhage, *n* (%)	37 (24.0)	12 (32.4)	0.293 ^a^	V = 0.08
Symptomatic intracranial hemorrhage, *n* (%)	15 (9.7)	7 (18.9)	0.149 ^a^	V = 0.11

Data are presented as *n* (%) for categorical variables and median [interquartile range, IQR] for continuous variables. ^a^ Pearson chi-square test or Fisher’s exact test (expected cell count < 5); ^b^ Mann–Whitney U test. ^g^ Effect size: Cramér’s *V* (φ) for categorical variables and *r* = |*Z*|/√*N* for continuous variables; values of approximately 0.10, 0.30 and 0.50 indicate small, medium, and large effects, respectively. Bold *p* values indicate statistical significance (*p* < 0.05). * Frailty was assessed using the 5-factor modified Frailty Index (mFI-5). ** Assessed only in patients with anterior circulation infarction and assessable baseline CT/CTA (*n* = 146; 118 aged 18–79 years and 28 aged ≥ 80 years). ASPECTS, Alberta Stroke Program Early CT Score; IV, intravenous; LKW, last known well; mRS, modified Rankin Scale; mTICI, modified Thrombolysis in Cerebral Infarction; NIHSS, National Institutes of Health Stroke Scale; NLR, neutrophil-to-lymphocyte ratio. Comparisons between age groups were univariable; effect modification by age was tested within the multivariable models (Supplementary Table S3).

## Data Availability

The data presented in this study are available on request from the corresponding author. The data are not publicly available owing to institutional and privacy restrictions.

## Supplementary Materials

The following supporting information can be downloaded at: https://www.mdpi.com/article/10.3390/medicina62102036/s1, Table S1: Sensitivity analyses of the final multivariable models for futile recanalization; Table S2: Comparison of patients with anterior circulation infarction and successful recanalization with and without assessable baseline imaging; Table S3: Exploratory analysis of effect modification by age (≥80 vs. 18–79 years) in the final multivariable models.

## Abbreviations

The following abbreviations are used in this manuscript:

AF	Atrial fibrillation
AIS	Acute ischemic stroke
ASPECTS	Alberta Stroke Program Early CT Score
AUC	Area under the curve
CI	Confidence interval
CKD	Chronic kidney disease
DM	Diabetes mellitus
ESMINT	European Society for Minimally Invasive Neurological Therapy
ESO	European Stroke Organisation
EVT	Endovascular therapy
FR	Futile recanalization
HT	Hypertension
ICH	Intracranial hemorrhage
IQR	Interquartile range
IV rtPA	Intravenous recombinant tissue plasminogen activator
LKW	Last known well
LVO	Large vessel occlusion
mFI-5	5-factor modified Frailty Index
mRS	Modified Rankin Scale
mTICI	Modified Thrombolysis in Cerebral Infarction
NIHSS	National Institutes of Health Stroke Scale
NLR	Neutrophil-to-lymphocyte ratio
OR	Odds ratio
ROC	Receiver operating characteristic
sICH	Symptomatic intracranial hemorrhage
STROBE	Strengthening the Reporting of Observational Studies in Epidemiology
TOAST	Trial of Org 10172 in Acute Stroke Treatment
