# Supplementary material for: Determinants of Futile Recanalization and the Influence of Age After Endovascular Therapy for Acute Ischemic Stroke: A Retrospective Single-Center Cohort Study
Source: Medicina (Kaunas). 2026 Oct 21;62(10):2036. doi: 10.3390/medicina62102036 (PMC13707255; doi:10.3390/medicina62102036)
Supplement: Supplementary file 1 [file medicina-62-02036-s001.zip › medicina-4567408-supplementary.pdf]

Supplementary Materials

Supplementary Table S1. Sensitivity analyses of the final multivariable models for futile recanalization.

	OR (95% CI)	*p*	OR (95% CI)	*p*	OR (95% CI)	*p*
**A. Model 1 variables**	**All patients (n = 191)**		**Anterior circulation only (n = 171)**		**Model 2 sample (n = 146)**	
Baseline NIHSS score (per 1-point increase)	1.29 (1.18–1.42)	**<0.001**	1.26 (1.14–1.39)	**<0.001**	1.34 (1.20–1.50)	**<0.001**
Baseline glucose (per 1 mg/dL increase)	1.01 (1.00–1.02)	**0.001**	1.01 (1.00–1.02)	**0.009**	1.01 (1.00–1.02)	**0.025**
Puncture-to-recanalization time (per 1-minute increase)	1.02 (1.00–1.03)	**0.036**	1.02 (1.00–1.03)	**0.040**	1.01 (1.00–1.03)	0.105
**B. Model 2 variables**	**Complete-case analysis (n = 146)**		**Multiple imputation (n = 171)**			
Dyslipidemia	3.51 (1.21–10.18)	**0.021**	2.80 (1.09–7.23)	**0.033**		
Baseline NIHSS score (per 1-point increase)	1.30 (1.15–1.47)	**<0.001**	1.21 (1.09–1.35)	**<0.001**		
Sufficient collateral status (vs. insufficient)	0.19 (0.08–0.47)	**<0.001**	0.17 (0.07–0.40)	**<0.001**		
ASPECTS (per 1-point increase)	0.60 (0.39–0.91)	**0.017**	0.61 (0.40–0.93)	**0.022**		
NLR (per 1-unit increase)	1.14 (1.02–1.28)	**0.017**	1.09 (0.99–1.18)	0.065		
Puncture-to-recanalization time (per 1-minute increase)	1.02 (1.00–1.05)	**0.019**	1.03 (1.01–1.05)	**0.008**		

(A) The final Model 1 (baseline NIHSS score, baseline glucose and puncture-to-recanalization time) refitted in all patients with successful recanalization (main analysis), in patients with anterior circulation infarction only, and in the Model 2 sample (anterior circulation infarction with assessable imaging). (B) The final Model 2 fitted in patients with complete imaging data (main analysis) and in all patients with anterior circulation infarction after multiple imputation of ASPECTS and collateral status by chained equations (predictive mean matching; 40 imputed data sets) with estimates pooled using Rubin's rules. Bold *p* values indicate $p < 0.05$. ASPECTS, Alberta Stroke Program Early CT Score; CI, confidence interval; NIHSS, National Institutes of Health Stroke Scale; NLR, neutrophil-to-lymphocyte ratio; OR, odds ratio.

Supplementary Table S2. Comparison of patients with anterior circulation infarction and successful recanalization with and without assessable baseline imaging (ASPECTS and collateral status).

	Imaging available (n = 146)	Imaging not available (n = 25)	*p* value	Effect size[g]
Futile recanalization, n (%)	88 (60.3)	16 (64.0)	0.724[a]	V = 0.03
Age, years, median [IQR]	72 [18]	67 [14]	0.539[b]	r = 0.05
Age ≥80 years, n (%)	28 (19.2)	6 (24.0)	0.591[a]	V = 0.04
Sex, male, n (%)	72 (49.3)	12 (48.0)	0.903[a]	V = 0.01
Hypertension, n (%)	90 (61.6)	14 (56.0)	0.593[a]	V = 0.04
Dyslipidemia, n (%)	43 (29.5)	8 (32.0)	0.797[a]	V = 0.02
Atrial fibrillation, n (%)	42 (28.8)	4 (16.0)	0.183[a]	V = 0.10
Frailty, n (%)	49 (33.6)	4 (16.0)	0.079[a]	V = 0.13
NIHSS score, median [IQR]	15 [6]	14 [6]	0.337[b]	r = 0.07
NLR, median [IQR]	3.08 [4.28]	8.60 [5.54]	**<0.001**[b]	r = 0.40
Glucose, mg/dL, median [IQR]	128 [46]	141 [72]	**0.024**[b]	r = 0.17
Creatinine, mg/dL, median [IQR]	0.90 [0.28]	0.93 [0.32]	0.445[b]	r = 0.06
LKW-to-puncture time, min, median [IQR]	280 [176]	352 [115]	**0.021**[b]	r = 0.18
Puncture-to-recanalization time, min, median [IQR]	47 [28]	40 [28]	0.190[b]	r = 0.10
mTICI 3, n (%)	127 (87.0)	25 (100.0)	0.079[a]	V = 0.15

	Imaging available (n = 146)	Imaging not available (n = 25)	*p* value	Effect size[g]
In-hospital mortality, n (%)	47 (32.2)	5 (20.0)	0.221[a]	V = 0.09

Data are presented as n (%) or median [interquartile range]. [a] Pearson chi-square test or Fisher's exact test (expected cell count <5); [b] Mann–Whitney *U* test. [g] Cramér's V (ϕ) for categorical variables and r = |Z|/√N for continuous variables. Bold *p* values indicate $p < 0.05$. Imaging was not available because baseline CT/CTA images could not be retrieved from the archive for review. LKW, last known well; mTICI, modified Thrombolysis in Cerebral Infarction; NIHSS, National Institutes of Health Stroke Scale; NLR, neutrophil-to-lymphocyte ratio.

Supplementary Table S3. Exploratory analysis of effect modification by age (≥80 vs. 18–79 years) in the final multivariable models.

Interaction term	*p* for interaction	Age-stratified or adjusted OR (95% CI)
**Model 1 (n = 191)**		
Baseline NIHSS score × age group	0.887	
Baseline glucose × age group	**0.011**	18–79 years: 1.02 (1.01–1.03); ≥80 years: 0.99 (0.98–1.01)
Puncture-to-recanalization time × age group	0.498	
Age ≥80 years (main effect, adjusted)	—	1.35 (0.56–3.28); *P* = 0.503
**Model 2 (n = 146)**		
Dyslipidemia × age group	0.522	
Baseline NIHSS score × age group	0.078	
Collateral status × age group	0.242	
ASPECTS × age group	0.111	
NLR × age group	0.731	
Puncture-to-recanalization time × age group	0.331	
Age ≥80 years (main effect, adjusted)	—	0.55 (0.16–1.81); *P* = 0.322

Age group and its product term with each retained predictor were added to the final models; *p* values for interaction were obtained with likelihood-ratio tests. Age-stratified ORs for glucose are per 1 mg/dL increase and were derived from Model 1 fitted separately in each age group. Given the small number of patients aged ≥80 years (n = 37 in Model 1; n = 28 in Model 2), these analyses are exploratory. ASPECTS, Alberta Stroke Program Early CT Score; CI, confidence interval; NIHSS, National Institutes of Health Stroke Scale; NLR, neutrophil-to-lymphocyte ratio; OR, odds ratio.
